# Neuronal‐spiking‐based closed‐loop stimulation during cortical ON‐ and OFF‐states in freely moving mice

**DOI:** 10.1111/jsr.13603

**Published:** 2022-06-03

**Authors:** Martin Kahn, Lukas B. Krone, Cristina Blanco‐Duque, Mathilde C. C. Guillaumin, Edward O. Mann, Vladyslav V. Vyazovskiy

**Affiliations:** ^1^ Department of Physiology Anatomy and Genetics, University of Oxford Oxford UK; ^2^ Sleep and Circadian Neuroscience Institute University of Oxford Oxford UK; ^3^ University Hospital of Psychiatry and Psychotherapy University of Bern Bern Switzerland; ^4^ Centre for Experimental Neurology University of Bern Bern Switzerland; ^5^ Nuffield Department of Clinical Neurosciences University of Oxford Oxford UK; ^6^ Department of Health Sciences and Technology Institute for Neuroscience ETH, Zurich Switzerland

**Keywords:** closed‐loop stimulation, cortical layers, mice, neocortex, sleep, sleep homeostasis, slow wave detection

## Abstract

The slow oscillation is a central neuronal dynamic during sleep, and is generated by alternating periods of high and low neuronal activity (ON‐ and OFF‐states). Mounting evidence causally links the slow oscillation to sleep's functions, and it has recently become possible to manipulate the slow oscillation non‐invasively and phase‐specifically. These developments represent promising clinical avenues, but they also highlight the importance of improving our understanding of how ON/OFF‐states affect incoming stimuli and what role they play in neuronal plasticity. Most studies using closed‐loop stimulation rely on the electroencephalogram and local field potential signals, which reflect neuronal ON‐ and OFF‐states only indirectly. Here we develop an online detection algorithm based on spiking activity recorded from laminar arrays in mouse motor cortex. We find that online detection of ON‐ and OFF‐states reflects specific phases of spontaneous local field potential slow oscillation. Our neuronal‐spiking‐based closed‐loop procedure offers a novel opportunity for testing the functional role of slow oscillation in sleep‐related restorative processes and neural plasticity.

## 1 INTRODUCTION

The possibility of non‐invasive modulation of sleep oscillations has recently attracted significant attention (Bellesi et al., 2014; Choi et al., 2020; Fattinger et al., 2019; Frase et al., 2019; Geiser et al., 2020; Krugliakova et al., 2022; Malkani & Zee, 2020; Marshall et al., 2006; Ngo et al., 2013; Schneider et al., 2020). Slow waves are a predominant type of sleep oscillatory activity during non‐rapid eye movement (NREM) sleep, but can also occur during rapid eye movement (REM) sleep and wakefulness (Andrillon et al., 2021; Bernardi et al., 2019; Borbely et al., 1984; Funk et al., 2016; Vyazovskiy et al., 2011, 2014). Sleep slow waves are homeostatically regulated (Achermann et al., 1993; Borbély, 1982; Huber et al., 2000; Krone et al., 2021; Thomas et al., 2020), and have been implicated in synaptic plasticity, metabolic restoration, glymphatic clearance and other functions (Frank & Heller, 2019; Krueger et al., 2016; Vyazovskiy & Harris, 2013). Traditionally, online detection of slow waves relies solely on their cortical surface‐ or scalp‐recorded electroencephalogram (EEG) waveforms (Moreira et al., 2021; Ngo et al., 2013; Santostasi et al., 2016), where the specific phase is assumed to correspond to periods of high or low neuronal activity (ON‐ and OFF‐states) or transitions between population activity and silence (McKillop et al., 2018; Nir et al., 2011). However, in such studies no attempts have been made to directly target the underlying neuronal network activity itself.

The central aim of this study was to develop and validate the methodology for online detection of ON and OFF periods, and to investigate the possibility of neuronal‐spiking‐based closed‐loop stimulation during spontaneous sleep in mice. The potential applications of this method include addressing the following questions.

1. The role of sleep in synaptic plasticity. In vitro evidence and experiments in anaesthetised animals suggest that pairing synaptic inputs with population ON and OFF periods leads to plastic changes in neural responses to stimulation (Bartram et al., 2017; Gonzalez‐Rueda et al., 2018). This observation is important, as it suggests that a careful choice of the phase of stimulation could make sleep more restorative but, alternatively, could also lead to sleep disruption and potentially to the development of maladaptive plastic changes within the thalamocortical circuitry. To this end, a better understanding of the role of ON and OFF periods in neural plasticity, as suggested by previous work, is essential.
2. Effects of ON/OFF‐states during spontaneous sleep on sensory responsiveness and processing of incoming stimuli (Massimini et al., 2005; Nir et al., 2015, 2017; Vyazovskiy, Faraguna, et al., 2009). We argue that this is critical, for example, to develop the most efficient and least disruptive stimulation protocols, and to establish whether the properties of induced slow waves differ depending on background activity.
3. Correspondence between neuronal activity and local field potential (LFP) waveforms. Finally, given that individual EEG slow waves vary greatly with respect to their origin, shape, amplitude and spatio‐temporal dynamics (Bukhtiyarova et al., 2019; Massimini et al., 2004; Murphy et al., 2009; Nir et al., 2011; Riedner et al., 2011), targeting those directly with conventional closed‐loop paradigms likely leads to many instances when stimulation is delivered during a suboptimal or even undesirable phase of the network oscillation. Arguably, this could influence the outcome of modulation. Therefore, obtaining a better understanding of the correspondence between neuronal activity and EEG/LFP waveforms across cortical layers will provide important refinement, both conceptual and methodological, for the approach used to target sleep slow waves.

## 2 METHODS

All experiments were carried out in accordance with the UK Animals (Scientific Procedures) Act of 1986. All animals used in this study were C57BL/6JOlaHsd purchased from Harlan Laboratories and kept on a regular (non‐inversed) 12 hr light/dark cycle. Seven male adult C57BL/6 mice (age at baseline recording 125 ± 8 days, body weight: 29.5 ± 0.8 g) were used for all experiments.

### 2.1 Implants and surgical procedure

All implants were prepared manually before the surgery. For the frontal and occipital EEG recordings, silver wires were wrapped around blunted skull screws and soldered to a 90‐degree connector (Pinnacle Technology, Lawrence). For the electromyogram (EMG), the end of a silver wire was bent into a U‐shape and then twisted, to avoid sharp edges. This was done on two separate wires that were soldered to the above‐described EEG head stage. The laminar probe (NeuroNexus Technologies; A1 × 16‐3 mm‐100‐703‐Z16) has a ground and reference wires, each soldered to male connector pins, which could then be connected during surgery to female connector pins on the ground and reference screw, respectively. The laminar probe was stained with the dye DiI (DiIC18[3], Invitrogen) before surgery to aid the localisation of the electrode tract (Krone et al., 2021).

**FIGURE 1:**
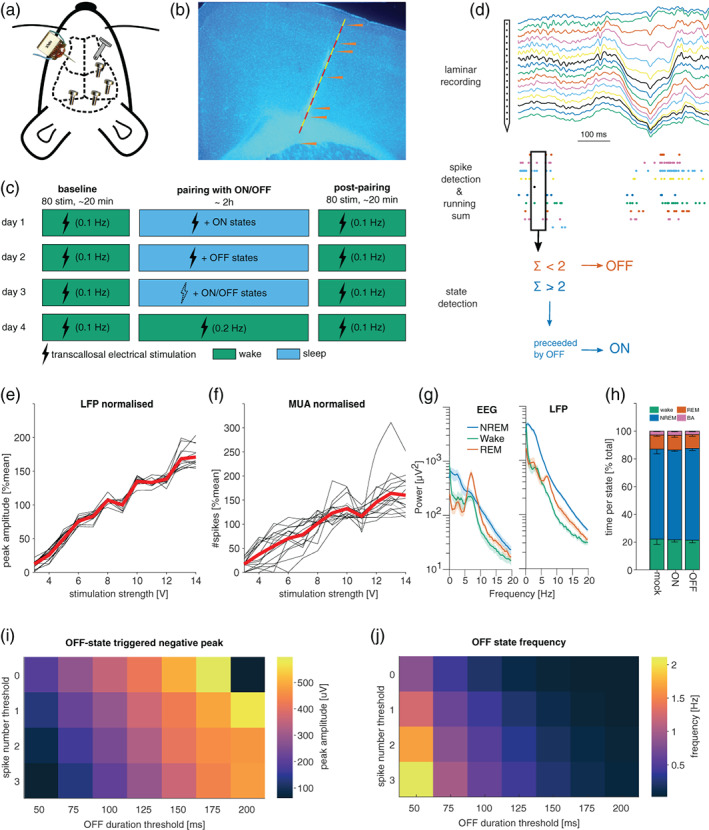
Methodological approach. (a) Schematic of cranial electrode placement: 16‐channel laminar probe (left) was inserted into M1 contralateral to the stimulation electrode (right), in addition to electroencephalogram (EEG) electrodes in the frontal and the occipital derivation. (b) Representative histology image. (c) Experimental design. Each mouse was subjected to four stimulation paradigms, each on a separate experimental day. Each stimulation paradigm includes a baseline and post‐pairing stimulation period with 80 stimulations at 0.1 Hz during wakefulness. (d) The method used for ON and OFF periods detection. (e) Example of dose–response curves in the local field potential (LFP) in response to stimulation in one animal. Black lines show the average response across trials of a single channel in the laminar probe. The red line is the average across all channels. (f) Same as (e), but for spiking. (g) Example of power spectral density in the EEG (left) and LFP (right) during different vigilance states. (h) Time spent per vigilance state (± SEM) across all mice in the different experimental conditions. (i,j) Simulations illustrating the effects of varying parameters of the real‐time ON/OFF‐state detection algorithm. Longer and stricter (i.e. fewer spikes) neuronal silent periods result in a larger peak in the LFP (i), which occur progressively more rarely (j). Note the resulting trade‐off between parameters resulting in a large LFP peak and parameters resulting in frequent detection events

To induce anaesthesia, the mouse was exposed to a prefilled chamber with 4% isoflurane in medical oxygen and, once the mouse had lost the righting reflex and approached a breathing rate of approximately 80 min^−1^, the animal was then transferred to a heating pad and 2–3% isoflurane administered through a nose mask at an oxygen flow rate of ~1–1.5 L min^−1^. After the scalp was shaved and cleaned using iodine and ethanol, the animal was transferred to a stereotaxic frame where isoflurane was administered at a concentration of 0.6–1.2% at a flow rate of ~1 L min^−1^ throughout the surgery. At this point, Metacam® (meloxicam, 5 mg kg^−1^; Boehringer Ingelheim), Vetergesic® (buprenorphine, 0.1 mg kg^−1^; Sogeval UK) and dexamethasone (0.2 mg kg^−1^ s.c.; Boehringer Ingelheim) were injected subcutaneously and artificial tears were applied. Once the head was fixed, a rectal probe was inserted to maintain core temperature at about 37°C. The scalp was opened, and the straightness of the skull was verified by levelling bregma and lambda, and the points 1 mm lateral to bregma. To minimise the loss of implants, the skull's surface was roughened using the scalpel and etching gel, and the coordinates for implantation were marked as shown in Figure 1(a). The holes for the reference (cerebellum), ground (cerebellum or left occipital) and the two EEG screws (frontal and occipital) were drilled first, and the screws were then immediately inserted using a screwdriver. Subsequently, the hole for the bipolar concentric stimulation electrode (Plastics One; see Section 2.3 “*Experimental design*” below for further information) was drilled, and the electrode was carefully and slowly inserted. All screws were then fixed with dental cement SuperBond® (Prestige Dental Products) before a craniotomy was made for the laminar electrode. Once the bone was removed, the dura was carefully rolled back with a syringe tip and the laminar probe was immediately inserted until the last of its 16 contacts was below the cortical surface (Figure 1b). The craniotomy was immediately sealed with a silicone gel (KwikSil; World Precision Instruments). The entire head stage was then cemented and the EMG wires inserted into the neck before the skin was sutured, if necessary. Animals were given subcutaneous saline injections following the surgery. After surgery, animals were carefully monitored at least once a day for 7 days, and analgesics were administered orally or subcutaneously, if necessary.

### 2.2 Electrophysiological recordings

Animals were moved to recording chambers at least 3 days before the start of any recording. At least 1 day into this habituation phase was allowed before the EEG head stage was connected to a cable bridging the animal and the pre‐amplifier, and another day before the laminar probe was connected to the pre‐amplifier. EEG and EMG signals were routed via an S‐box (Tucker Davies Technologies [TDT], Alachua, FL, USA) to a PZ‐5 pre‐amplifier (TDT), where they were differentially digitised (relative to the cerebellar screw or the contralateral EMG wire, respectively) at 25 kHz. The signal was then sent to a RZ‐2 signal processing system (TDT), which interfaced with the synapse recording software (TDT). The RZ‐2 sampled the signal down to 700 Hz (applying an adequate low‐pass filter at 45% of the final sampling frequency) and stored it at 305 Hz. Whenever possible, the signal was stored in this “raw” form in addition to versions with high pass filters more suitable for online monitoring (i.e. 0.5 and 10 Hz for EEG and EMG, respectively).

The signal from the laminar probe was routed directly to the PZ‐5 and sampled at 50 kHz. To obtain continuous LFP data (and limit data size), one version of the signal was down sampled to 305 Hz identical to the EEG signal. For stimulation‐evoked activity, a snippet of the LFP was stored at 3.5 kHz whenever the recording software triggered a stimulation. Specifically, the snippet started 500 ms before the stimulation and was 1.5 s long. An important consequence of this procedure is that there may be a small (< 1 ms) delay between the time when the software sends out the trigger and when the current is applied by the stimulation box.

To record spiking activity, the laminar signal (at 25 kHz) was filtered between 300 and 3000 Hz, and a manual threshold was set for each channel individually. The manual threshold was initially set at least 2 standard deviations from the mean. It was then further increased until the average spike waveform (10 s running window) no longer contained noise. Whenever the signal on a given channel crossed the threshold, the timestamp of threshold crossing and a 1.56‐ms‐long snippet of the signal was stored at 12 kHz. This procedure has the advantage that it strongly reduces the considerable data load of recording 16 channels for days at 25 kHz. On the other hand, it irrevocably discards data, especially given than each channel typically recorded spiking activity from more than one individual neuron. In other words, some spikes are too small to trigger the threshold, while some noisy events or spikes produced by one neuron will trigger it and thus create a 1.56‐ms‐long refractory period where spikes produced by other neurons will be lost. Spiking activity was always processed with WaveClus (Chaure et al., 2018). This software was chosen because it was designed explicitly to perform well on single‐channel recordings as well as multi‐channel recordings. In contrast, most other spike‐sorting algorithms are optimised for polytrode recordings (Chung et al., 2017), where a single unit is recorded on > 1 channel. Such cases are likely rare in the present recordings, given the relatively large distance between channels (100 μm).

### 2.3 Experimental design

Our experimental design included cortical electrical stimulation during both waking and sleep to investigate: (a) the immediate effects of stimulation on cortical responses; and (b) to address the effects of stimulation on synaptic plasticity (Bartram et al., 2017; Vyazovskiy et al., 2008, 2013) To this end, every mouse was subjected to at least four basic experimental conditions on separate days (Figure 1c). Each condition began with 80 stimulations at 0.1 Hz (the current pulses were 0.1‐ms squared monophasic pulses and the chosen output voltage was normally about 6–10 V) approximately at ZT 1 (1 hr after lights on). During this “baseline waking” period, mice were kept awake by providing novel objects. Following this baseline stimulation, animals were exposed to the different experimental conditions (described below) for approximately 2.5 hr. This will henceforth be referred to as the “pairing” period, because electrical stimulation was typically paired with a specific state (even though no stimulation may occur in some cases). After this pairing period, a post‐pairing wake stimulation followed in all conditions. This post‐pairing wake stimulation was always identical to the pre‐pairing wake stimulation on all days for the same mouse (very subtle differences in baseline stimulation parameters occur in a few mice, but all variance is between mice, never within mice). As shown in Figure 1(c), the four basic conditions were: (1) sleep‐mock: stimulation was targeted alternately at ON/OFF‐states but the stimulation box was turned off; (2) sleep‐ON; and (3) sleep‐OFF where stimulation was targeted selectively at ON‐ and OFF‐states, respectively; (4) wake‐stim: the same number of stimuli were delivered as during (2) and (3), but the animal was kept awake with novel objects. The interstimulus interval was similarly constrained as during (2) and (3), but was adjusted such that the same number of stimuli was delivered in approximately the same amount of time. The number of stimulations delivered during these pairing protocols was determined by the first experimental day in each animal. The animal was allowed to sleep for up to 2.5 hr, and the only constraint on stimulation numbers was the minimum interstimulus interval (10 s), the amount of NREM sleep and the number of ON/OFF detections. Thus, the interstimulus interval was sufficiently long to prevent induction of plasticity or over‐stimulation, but also sufficiently short to obtain a sufficient number of stimulations for subsequent analysis. The total number of stimulations during the pairing period varied slightly between animals but never within animals (i.e. it never varied between conditions). In all subsequent experimental days, the same number of stimulations was delivered (except for mock stimulation days). Therefore, the total duration of the experiment was kept constant at approximately 2.5 hr, but varied slightly (within ~20 min) between conditions. To avoid a systematic effect of repeated stimulation, the order of the conditions was randomised, except that wake‐stim (4) was never done as the first condition, because the number of stimuli delivered during the pairing period was constrained most strongly by the ON/OFF detection algorithm. The stimulation strength was chosen based on the dose–response curve (Figure 1e,f). The stimulation strength was then set as the weakest stimulation strength sufficient to elicit a measurable response.

### 2.4 Online ON and OFF period detection, and closed‐loop electrical stimulation

Procedures for online data processing and closed‐loop stimulation were custom written in the proprietary object‐oriented programming environment supplied by TDT and summarised in Figure 1(d). First, the incoming spikes were summed across all channels over a predefined time window (usually 50–125 ms). Whenever this running sum went below a predefined threshold (usually 1 or 2 spikes), an OFF‐state was registered. An ON‐state was defined as a period of high firing (10–30 Hz) for a prolonged period of time (same duration as OFF‐state), following an OFF‐state. To avoid stimulating during waking, a running root mean squared of the EMG signal was used and a manual threshold was set for it. Electrical stimulation was delivered to the animal through a bipolar concentric stimulation electrode attached to a PSI6x stimulus isolation unit. The stimulus isolation unit was coupled to a stimulation box (Grass Instruments), on which the stimulation parameters could be set manually. Once the parameters were set, the stimulation box could be triggered by means of a transistor‐transistor logic pulse from the RZ‐2 system, which was controlled by the recording software. One day before experiments started, an input–output curve was obtained (Figure 1e,f). The 1.5‐s‐long LFP snippets (sampled at 3–6 kHz) surrounding each stimulation were imported into Matlab using the supplier's (TDT) Matlab software developing kit. Pre‐processing of the snippets involved removing line noise and slow drift using a regression‐based algorithm (http://chronux.org/; Mitra & Bokil, 2009). Specifically, we used the Chronux function *locdetrend*, which applies a least‐squares fit to a running window (800 ms width, 100 ms steps). Regression‐based approaches were chosen to avoid introducing filtering artefacts. For analysis of the peak and slope of the evoked response, potential direct current‐offsets were accounted for by subtracting the mean of the 5 ms preceding the stimulation from the entire snippet for each channel and trial separately.

### 2.5 Histology

After the experiments were completed, animals were deeply anaesthetised with an intraperitoneal injection of pentobarbital (Euthanal). Once the animal reached deep anaesthesia (as verified by loss of righting, pedal and corneal reflexes), microlesions were performed to aid laminar identification of recording sites (Krone et al., 2021). For microlesions, the laminar probe was connected to an impedance testing device (NanoZ, Plexon), which was used to pass current (10 μA for 10 s) through four equally spaced channels of the laminar probe. The bottom channel was always lesioned first, as the first lesion can damage the other channels. Animals were then transcardially perfused with phosphate‐buffered saline (PBS) and 4% paraformaldehyde (PFA), and the head of the animal was then stored in 4% PFA (i.e. the implant was not removed at this point, which improved the quality of histology) and moved into acidified PBS after a few days. Brains were embedded in agarose and cut into 50‐μm‐thick coronal sections. The sections were stained with 4′,6‐diamidino‐2‐phenylindole (DAPI) and imaged using a fluorescence microscope. The sections containing the electrode tract were identified using the red Dil fluorescence, and were imaged at 1.6, 2.5 and 5 × magnification. The recording locations in the rostrocaudal and mediolateral dimensions were identified using the mouse brain atlas (Paxinos & Franklin, 2001). The cortical layer of each laminar contact was identified in the 5 × magnification images. First, the site(s) of the microlesions were identified in the DAPI or background fluorescence (green fluorescent protein) images. Second, the position of lesions, the Dil staining and the length of the electrode were used to determine the position of each contact. Layer 1 was identified based on the low density of neurons compared with layers 2/3. Similarly, the beginning of layer 5 was identified based on the lower cell density in layer 5 and the presence of large pyramidal cells characteristic for this layer. While layer 4 is comparatively small in the primary motor cortex, it exists and can be identified as a small increase in cell density right above layer 5 (Skoglund et al., 1997; Yamawaki et al., 2014). Layer 6 was also identified based on the higher density of cells compared with layer 5.

### 2.6 Scoring of vigilance states

Data were extracted from the raw data format of the recording software, resampled to 256 Hz and bandpass‐filtered using custom Matlab scripts (0.5–100 Hz for EEG/LFP and 10–50 Hz for EMG, 3rd order phase conserving type II Chebyshev filter). The signals were then converted to the ASCII format and from there converted into European Data Format (EDF) files. The EDF files were visualised in the software SleepSign (Kissei Comtec, Nagano, Japan). To score vigilance states, the LFP, EEG and EMG data were examined in 4‐s epochs. If present, timing of electrical stimulation was also visualised. Waking was defined as a low‐voltage, high‐frequency EEG with a high variance in the EMG. In contrast, NREM sleep was defined as high‐amplitude EEG signals containing slow waves (and high delta power) and exhibiting low EMG tone and variance. The EMG commonly displayed clear heartbeat artefacts during all sleep episodes. REM sleep was defined as wake‐like activity with sleep‐like EMG signal and usually high theta activity in the occipital derivation (resulting EEG and LFP power spectra are shown in Figure 1g). When an animal displayed wake‐like activity for less than 4 consecutive epochs (i.e. 16 s) within a NREM bout, this was scored as brief awakening. Episodes of all four types (NREM, REM, waking, brief awakening) were flagged if they contained clear artefacts in any EEG or LFP channel. When sleep scoring was complete, the SleepSign software returned the vigilance states and the power spectra for each 4‐s episode; the latter were calculated in 0.25‐Hz frequency bins using a Hanning window. Special consideration was given to 4‐s epochs containing stimulation events. For stimulations aimed at waking periods, the epoch was only scored as NREM if there was sleep‐like activity in the 2 s before or after stimulation. Vice versa, if stimulation was aimed at NREM episodes, activity was scored as REM or waking if the activity 2 s before or after the stimulation resembled the respective state. The same “over‐sensitive” procedure was applied with regards to artefacts. We found that stimulation did not have a major effect on the amount of vigilance states, and > 95% of stimulations targeted sleep as intended.

### 2.7 Statistics

The experimental design of this study posed several statistical challenges. Most notably, each mouse experienced several treatments, and observations were often nested (e.g. multiple channels, per mouse and several mice per condition). To address these challenges, we used linear mixed effects (LME) models (Harrison et al., 2018). This method has several advantages, most notably it can account for the abovementioned nested nature of experiments and it can readily handle missing data points (e.g. a noisy or unresponsive channel on 1 day). Each time an LME was used, all assumptions of LMEs (independence, homogeneity of variance, normality of error, and linearity) were visually inspected using plots (e.g. QQ plots). To test for significance, we used Matlab and R‐studio to fit a model with and without the relevant parameter (e.g. condition) and compared the models using the log‐likelihood ratio (LLR) test. If the result was significant we ran post hoc Tukey contrast in R‐studio.

## 3 RESULTS

### 3.1 Real‐time detection of ON‐ and OFF‐states during sleep in freely moving mice

We chronically implanted seven mice with frontal and occipital screws to monitor the EEG, and with two wires in the neck muscles to measure the EMG. For neuronal activity recording, we implanted a 16‐channel laminar probe into the primary motor cortex (M1; Figure 1a). To detect ON‐ and OFF‐states online, spikes were summed across all channels of the laminar probe (Figure 1d). OFF‐states were detected when the running sum of spikes was below a certain threshold (usually below 1 or 2 spikes) for a sufficient amount of time (50–125 ms). An ON‐state was defined as a period of high firing (10–30 Hz) for a prolonged period of time (same duration as OFF‐state), following an OFF‐state. A challenge for this procedure is the trade‐off between speed and accuracy and the trade‐off between sensitivity and selectivity. Furthermore, the optimal parameters are not uniform across animals, in part due to different numbers of neurons recorded by each laminar probe. Therefore, we used a baseline recording of each mouse to simulate ON/OFF‐state detection with differing parameters. As expected, increasing the minimum duration of OFF/ON‐states leads to detection of larger amplitude slow waves in the LFP but also to fewer detections of ON/OFF‐states (Figure 1i,j), as has been previously reported (McKillop et al., 2018; Vyazovskiy, Olcese, et al., 2009). We surmise that increasing the minimal duration of OFF/ON‐states leads to an increased chance to detect a state towards its very end.

**FIGURE 2:**
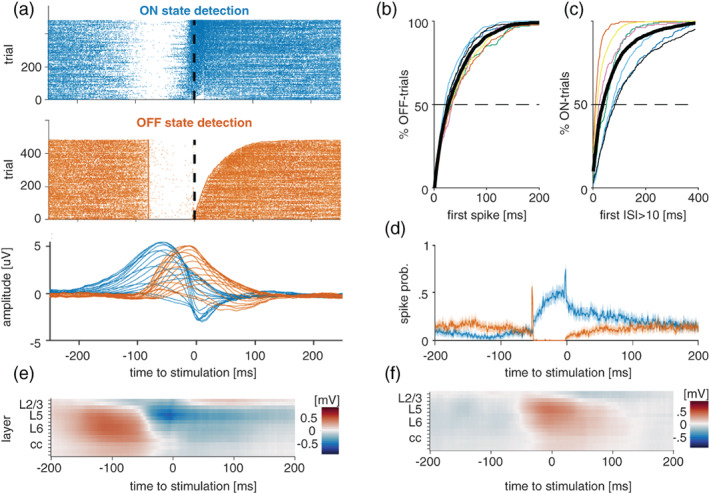
Online detection of ON/OFF‐states in freely moving mice. (a) Top: Example of all ON‐ and OFF‐state detections in one representative mouse during mock stimulation. Dashed line indicates when stimulation would be delivered. Bottom: Example of average ON/OFF‐state‐evoked local field potential (LFP) signal across all channels in the same animal as shown above. (b) Cumulative histogram of the time until the occurrence of the first spike following an OFF‐state detection. Thin coloured lines depict individual mice, thick black line corresponds to the mean across animals. (c) Cumulative histogram of the time until the occurrence of the first OFF‐state following an ON‐state detection. Thin coloured lines depict individual mice, black line is mean across animals. (d) Average ON/OFF‐state‐evoked spiking across all channels in one representative mouse. (e) Example of average LFP signal across all channels centred on ON‐state detections. (f) Example of average LFP signal across all channels centred on OFF‐state detections

As expected, we found that OFF‐state detection was always preceded by a period of neuronal quiescence, whereas ON‐state detections were preceded by increased spiking (Figure 2a). Upon detection of OFF and ON periods, the probability to transition out of the detected state began to increase logarithmically (Figure 2b,c). Importantly, the detection of ON and OFF periods based on neuronal spiking was on average associated with LFP slow waves (Figure 2a), and with expected changes in multiunity acitivity (MUA) (Figure 2d). A clear‐cut laminar profile of LFP signals associated with detected neuronal ON and OFF periods was apparent (Figure 2e,f), consistent with the notion that LFP slow waves and their underlying neural dynamics originate from the deep cortical layers (Beltramo et al., 2013; Krone et al., 2021; Sanchez‐Vives & McCormick, 2000).

### 3.2 Neuronal responsiveness differs between ON and OFF periods

**FIGURE 3:**
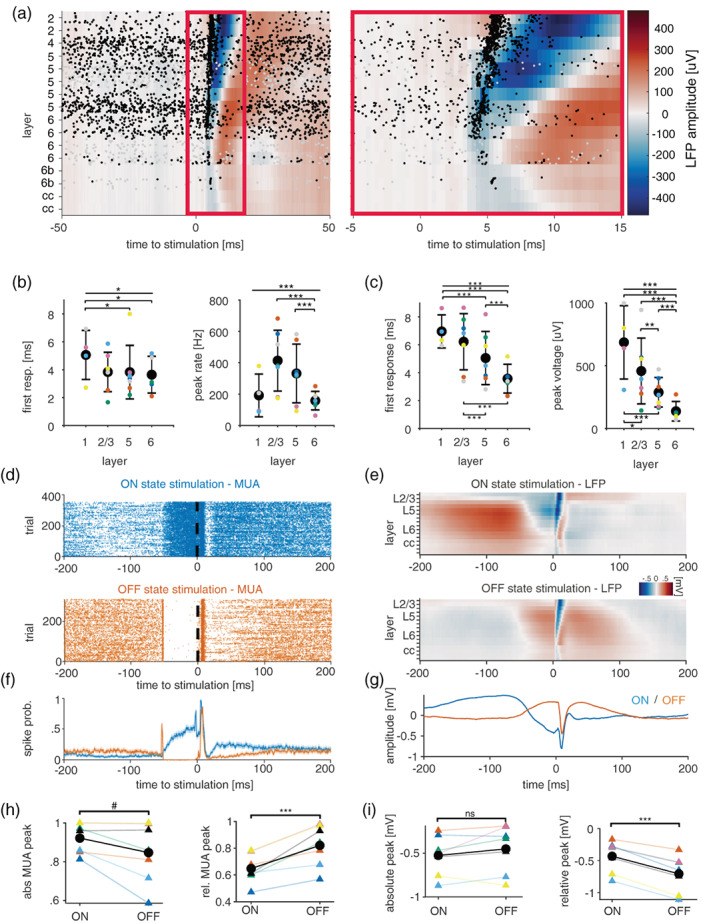
Modulation of evoked responses by stimulation during ON‐ or OFF‐states. (a) Laminar local field potential (LFP) and MUA responses to transcallosal electrical stimulation. Spike occurrences are shown as raster plots. (b,c) Characteristics of MUA and LFP responses, respectively, across cortical layers of M1. *p < 0.05, **p < 0.01, ***p < 0.001. (d,e) Representative examples of stimulation‐evoked MUA and LFP responses, respectively, during ON (top)‐ and OFF (bottom)‐states. (f,g) Example of average stimulation‐evoked MUA and LFPs, respectively, across all channels during ON‐ or OFF‐states (stimulation delivered at time 0). (h,i) Quantification of absolute (left) and normalised (right) stimulation‐evoked spike rates and LFP response, respectively. ^#^0.05 > *p* < 0.06, ***p < 0.001, paired *t*‐test

For each mouse, we established an input–output curve for electrical stimulation at least 1 day prior to experiments (Figure 1e,f), and selected the weakest stimulation level that evoked a detectable response in both the MUA and the LFP. We first examined the LFP and MUA response to contralateral stimulation across cortical layers during artefact‐free wakefulness epochs (Figure 3a–c). Significant spiking responses (permutation test with 5000 permutations) to electrical stimulation occurred with an average probability of 51 ± 18% (mean ± SD, *n* = 7 mice with 16 channels each) across layers 1, 2/3, 5, and there was a significant effect of layer on response probability (*p* < 0.001, LLR test [dF = 3, χ^2^:37.9]). The spiking response generally involved a period of increased firing, followed by a period where spike rates fell below the spontaneous rates. The increased firing rate began on average 3.66 ± 0.93 ms (mean ± SD; *n* = 72 channels from 7 mice) after stimulation, and started significantly later in layer 1 compared with L5 and L6 (Figure 3b). Notably, in every experiment there was at least one channel that significantly responded within 1 ms of stimulation (mean time to first responsive time bin in any channel across mice: 1.59 ± 0.69 ms [mean ± SD]). This could be due to unaccounted stimulation‐induced noise or antidromic activation. The spiking response peaked between 5 and 10 ms and, on average, lasted until 10.7 ± 2.13 ms (mean ± SD) after the stimulus.

The LFP response was closely related to the spiking response, but appeared to be slightly delayed. Averaged across all responsive channels, the LFP had a negative peak of 314 ± 172 μV at 8.7 ± 1.85 ms after the stimulation (mean ± SD; *n* = 7 mice with 16 channels each). The LFP response began (defined here as crossing 2 SDs of pre‐stimulation baseline) on average 5 ± 1.77 ms after the stimulation and lasted for 8.2 ± 1.9 ms (mean ± SD, *n* = 90 channels from 7 mice), which is consistent with the durations of cortical postsynaptic potentials recorded in single neurons. The anatomical layer had a similar but clearer influence on the LFP compared with the MUA. Similar to the MUA, responsiveness of channels declined with depth, with layer 6 being significantly less likely to respond than all other layers (Figure 3c). Similarly, deep layers had smaller negative peaks, with almost all layer‐wise comparisons confirming this pattern. Despite having small peaks, deeper layers tended to respond and peak considerably earlier than superficial layers. Taken together, the layer profile of the evoked response in the MUA and LFP are consistent with a scenario where synaptic inputs reach deep layers first and then reach higher layers through cortico‐cortical transmission.

We next examined the evoked responses to ON/OFF stimulation during NREM sleep (Figure 3d–i). The prediction from several previous studies in anaesthetised animals and brain slices is that the magnitude of the response should be significantly modulated by ON/OFF‐states (Haider et al., 2007; Reig et al., 2015). In line with this, we find that the stimulation‐triggered increase in spiking (i.e. relative to pre‐stimulation baseline) is larger during OFF‐state pairings compared with ON‐state pairings (*p* = 0.0024, paired *t*‐test, *n* = 7). However, when the baseline is not subtracted the opposite trend emerges, with responses during ON‐states displaying a larger absolute peak (*p* = 0.0676, paired *t*‐test, *n* = 7; Figure 3h). A similar pattern emerges in the LFP response: when the baseline difference at stimulation onset is accounted for, the response is larger during OFF‐state stimulation. If it is not, there is no longer any evidence for a difference (Figure 3i).

### 3.3 Effects of electrical stimulation on sleep architecture and slow‐wave activity (SWA)

We next asked whether and how stimulation targeting ON and OFF periods affects sleep. This is relevant because if stimulation during ON‐ and/or OFF‐states has an immediate effect on sleep (e.g. waking the animal up), then this would be a confound for interpreting the effect of stimulations. However, as shown in Figure 1(h), there was no evidence for an effect of stimulation on the relative time spent in NREM sleep, REM sleep or awake (*n* = 7 mice, three separate repeated‐measures ANOVA, effect of pairing condition on % NREM with sphericity assumed: *p* = 0.518, *F*_2,12_ = 0.695; wake: *p* = 0.251, *F*_2,12_ = 1.555; or REM [Friedman's test, *p* = 0.180]).

**FIGURE 4:**
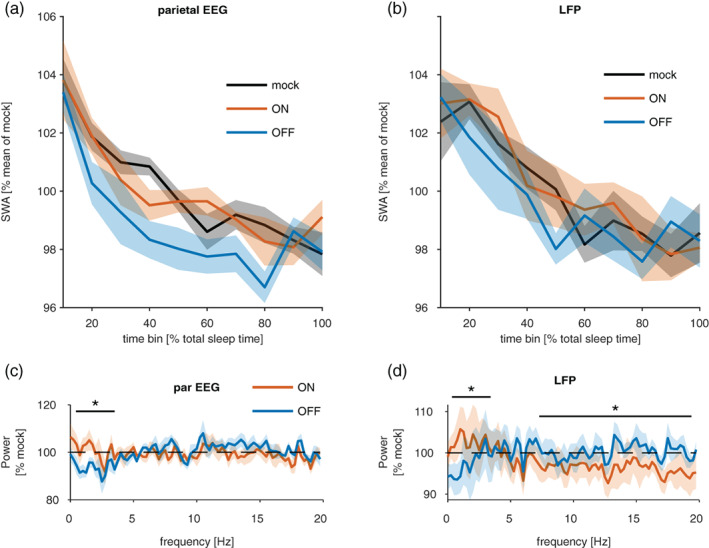
Effects of closed‐loop stimulation on sleep electroencephalogram (EEG). (a,b) The time course of EEG slow‐wave activity (SWA; 0.5–4 Hz) during non‐rapid eye movement (NREM) sleep across the stimulation session in the parietal EEG signal and the local field potential (LFP) recorded from M1 area. Relative values of SWA are plotted in 10‐min bins separately for mock stimulation, ON and OFF pairing conditions. Mean values ± SEM, *n* = 7 mice. (c,d) The effects of stimulation on EEG and LFP power spectra during NREM sleep. Spectral power values in ON and OFF pairing conditions are expressed as percentage of corresponding frequency bins during the mock stimulation condition for the parietal EEG and LFP signals. Asterisks above denote frequency bands where the effects of ON and OFF pairing differed significantly (*p* < 0.05)

To test whether stimulation has immediate or delayed effects on the progression of SWA across sleep, we assessed the time course of SWA in the EEG and LFP using only epochs not containing a stimulation event (Figure 4a). As expected, homeostatic decline of SWA resulted in a significant main effect of time on SWA in LME models run separately for the EEG and the average SWA in the LFP (LLR, χ^2^(9) = 112.9 and 147.9 for EEG and LFP, *p* < 10^−16^ for both, *n* = 7 mice). In addition, the parietal EEG displayed a significant effect of condition on SWA (χ^2^(2) = 21.62, *p* = 10^−5^), whereas there was no such effect of condition on SWA in the LFP (χ^2^(2) = 2.7, *p* = 0.25). There was no evidence for an interaction between condition and time in the EEG/LFP (LLR, χ^2^(18) = 16/12, *p* = 0.59/0.8). Post hoc comparisons in the EEG suggested that OFF‐state pairings were associated with significantly reduced SWA, compared with ON‐state pairings and mock pairings (Tukey contrasts, *p* < 10^−5^ for both comparisons in the EEG). This suggests that, independent of when the measurement was taken, the OFF‐state pairing condition always displays lower SWA. This contrasts slightly with the visual impression that the first and last time bins are not different, and is likely due to insufficient power.

The slow oscillation is not the only network phenomenon during natural NREM sleep, and other events, such as spindles, have been associated with plasticity. To test the effect of stimulation on other frequencies we calculated the difference between the average power spectra (again including only 4‐s epochs without stimulation) across conditions (Figure 4c,d). As expected from the previous results, there was a significant interaction between condition and frequency in the LFP (χ^2^(1) = 36.9, *p* = 10^−9^, LLR test) and in the frontal EEG (χ^2^(79) = 108.17, *p* = 0.016, LLR test). Post hoc test on individual 0.25‐Hz frequency bins suggested that EEG power in the frequencies between 0.25 and 2.5 Hz was lower during OFF‐ compared with ON‐state pairings, and not significantly different in other bands (pairwise contrasts without correction for multiple comparisons, bins with *p* ≤ 0.01: 0.75–2 Hz, all other significant bands are 0.05 > *p* > 0.01, estimated differences ranged from 7 ± 4.11% [2.5 Hz] to 12 ± 4.11% [1.25 Hz]). In the LFP (Figure 4d), there was also evidence that ON and OFF pairing had differential effects on power spectra (interaction between condition and frequency: χ^2^(79) = 685.8, *p* = 10^−16^, LLR test, LME models with channels and mouse as nested random effects). Post hoc tests for individual frequency bins indicated that sleep during ON‐state pairings had more power compared with sleep during OFF‐state pairings in low‐frequency bands (0–2 Hz, *p* < 0.001 for all bins except 1.5 Hz with *p* = 0.005), but it had lower power in several higher frequency bands (pairwise contrasts without correction for multiple comparisons, 5.25–5.75 Hz, *p* < 0.01; 7–8.25 Hz, *p* < 0.05; 9.25–9.75 Hz, *p* < 0.05; 10.5–20 Hz, *p* < 0.01) for most bins. Together, these data indicate that ON‐ and OFF‐state pairings have a differential effect on the power spectra of NREM episodes that do not contain a stimulation event. Interestingly this is the case for both the LFP and the EEG. Furthermore, this difference is likely driven by the OFF‐state pairings, which decrease several frequencies in the SWA range and increase (fewer) frequencies in the spindle range.

### 3.4 Using closed‐loop ON/OFF stimulation to estimate effect sizes of sleep‐dependent plasticity

One important application of the approach we describe here is to address the hypothesis that pairing an input to cortex with ON‐ and OFF‐states has differential effects on synaptic strength. To this end, we recorded LFP and neuronal responses to contralateral electrical stimulation in awake mice exploring objects, and used the magnitude of this response in the LFP and MUA as a proxy for synaptic strength (Fisher et al., 2016; Vyazovskiy et al., 2008; Vyazovskiy, Olcese, et al., 2009). We delivered 80 stimulations (0.1 Hz) before and after each of the following different pairing protocols shown in Figure 1(c): stimulation during ON‐states, stimulation during OFF‐states, mock stimulation (stimulation turned off), or during waking (novel objects were given to promote wakefulness when necessary).

**FIGURE 5:**
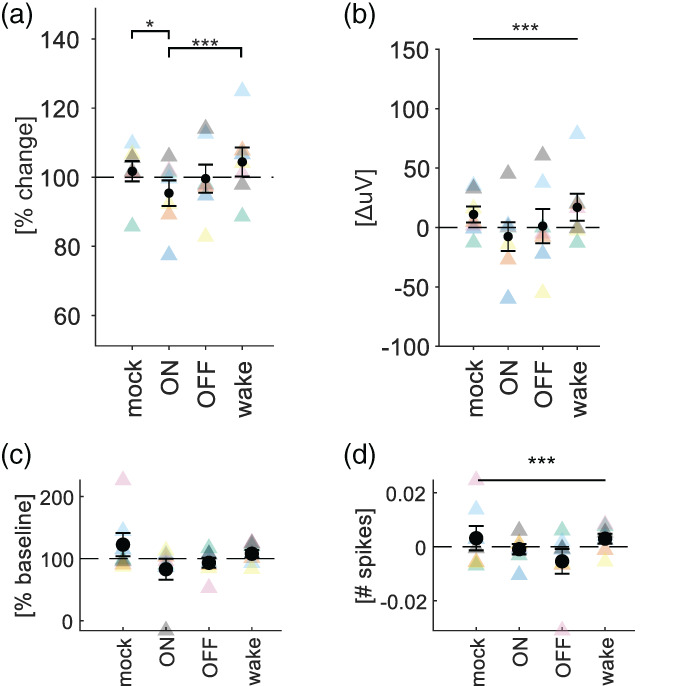
Effects of stimulation targeting ON and OFF periods during sleep on MUA and local field potential (LFP) responses during waking. (a) Changes in average LFP amplitude triggered by stimulation during wakefulness before and after pairing stimulation with ON‐ or OFF‐states. Peak responses were normalised by their baseline amplitude. Black circles show mean ± SEM, and coloured triangles show individual mice. Bars represent pairwise comparisons. *p < 0.05, **p < 0.01, ***p < 0.001. (b) Same as in (a) but without normalisation to baseline. Bars spanning all conditions indicate significant main effect of condition. ***p < 0.001. (c,d) Same as in (a) and (b), but for MUA

The effect of the four different pairing conditions (referred to as “condition”) on the change in LFP peak amplitude from pre‐ to post‐pairing wakefulness (Figure 5) was assessed with LME models of the form: $\Delta V=\text{condition}+V_{\text{baseline}}+\text{condition}*V_{\text{baseline}}+\left(1|\text{channel}:\text{mouse}\right)+\left(1|\text{mouse}\right)$.

The model supported a significant effect of condition on the change in LFP peak amplitude (Figure 5a). This was true for both the relative change (e.g. *V*_post_/*V*_pre_) and the absolute change (e.g. *V*_post_ − *V*_pre_; Figure 5ab). However, post hoc tests only yielded significant differences for the relative change, suggesting ON pairings are associated with a stronger decrease in amplitude compared with all other pairings save OFF‐state pairings (Tukey‐adjusted contrasts for difference in *β*‐values ± SE: ON‐mock: −7.2 ± 2.4%, *p* = 0.03; ON‐wake: −10.39 ± 2.4%, *p* < 0.001; ON–OFF: −4.71 ± 2.4%, *p* = 0.37; Figure 5a). There was no evidence for a significant interaction between baseline amplitude and condition. While wake pairings had a trend towards increasing the response, this was not significant. We applied the same statistical analysis to the neuronal firing rates (Figure 5c,d). In contrast to the LFP response, the model did not support an effect of stimulation on the relative change in response (Figure 5c). While the model supported an effect of condition on the absolute change in the number of spikes in response to stimulation (Figure 5d), no post hoc test was significant. Visual inspection of the data suggested that the condition with the biggest effect was pairing of stimulation with wakefulness.

In summary, our data suggest that different neuronal states have varying effects on neuronal plasticity. However, using the methodology in the present paper, the differences were subtle (about 5%), and would thus require a substantially bigger sample size to allow more robust conclusions.

## 4 DISCUSSION

Here we developed a method for online detection of cortical ON/OFF‐states during spontaneous sleep in freely‐behaving laboratory mice. While closed‐loop stimulation during slow waves is becoming increasingly popular, studies are typically based on the LFP or EEG signals only (Bellesi et al., 2014; Fattinger et al., 2019; Ngo et al., 2013; Schneider et al., 2020), which only indirectly reflect underlying network ON‐ and OFF‐states (Thomas et al., 2020; Timofeev, 2013). One biologically effective means for closed‐loop stimulation relies on setting an (adaptive) negative threshold to detect presumed OFF‐states, and then targeting stimuli into the subsequent UP state by using the average delay between positive and negative peaks for each individual (Ngo et al., 2013). This method affects memory (Ngo et al., 2013), changes SWA, and influences the immune system (Besedovsky et al., 2017). However, no studies until now have undertaken a direct online targeting of neuronal network ON‐ and OFF‐states during sleep.

Our key conclusion is that online targeting of ON and OFF periods based on spiking activity results in a reliable detection of specific phases of LFP slow waves. Our study is consistent with the existing knowledge that spontaneous LFP and EEG slow waves, on average, correspond to a general reduction or a complete cessation of neural spiking, reflecting population OFF periods. It should be noted, however, that sleep has properties of a local process (Krueger et al., 2008) and, arguably, the neocortex is never entirely in an ON or OFF period (Nir et al., 2011; Siclari & Tononi, 2017; Timofeev, 2013). Therefore, targeting a specific phase of a slow wave in one cortical region will be likely associated with targeting a different – and thus potentially suboptimal – phase in a different area of the brain. The consequences of such differential manipulations of slow waves in different cortical areas remain to be determined. We further observed that the evoked responses in the LFP began and peaked in deeper layers before the superficial layers. In the MUA, this trend was much less clear and likely present in only a subset of animals. An early response in deeper layers would be consistent with a strong innervation of layer 5 by callosal projections (Petreanu et al., 2007). However, such a pattern is also conceivable via polysynaptic pathways (i.e. contralateral M1 – region X – M1) and not least via antidromic activation. And yet, even if the laminar probe was placed perfectly in the area most strongly innervated by the stimulated area, or if only antidromic activation occurred, most of the recorded responses (particularly in the LFP) would likely be the consequence of local synaptic connectivity.

One potential application of our new method is to explore the role of sleep and associated patterns of population neuronal activity in synaptic plasticity, to which the current study provides initial, proof‐of‐principle results. Numerous studies demonstrated that cortical synaptic strength and firing activity are dynamically modulated across the day or, more precisely, as a function of sleep–wake cycle (Cirelli, 2017; Hengen et al., 2016; Seibt & Frank, 2019; Watson et al., 2016). Sleep was linked with strengthening of some synaptic connections (which is thought to mediate consolidation of long‐term memories; Chauvette et al., 2012), and weakening or elimination of others (de Vivo et al., 2017), which is thought to allow homeostatic rebalancing of net synaptic strength across the network (Watson et al., 2016). Evidence supporting the profound effects of sleep–wake states on synaptic plasticity includes LFP correlates, such as changes in slope of population synaptic response to stimulation (Chauvette et al., 2012; Vyazovskiy et al., 2008), neuronal activity (Fisher et al., 2016; Watson et al., 2016), phosphorylation status of receptors (Bruning et al., 2019; Diering et al., 2017; Noya et al., 2019; Vyazovskiy et al., 2008) and ultrastructural evidence obtained using electron microscopy (de Vivo et al., 2017).

It is not yet clear whether changes in firing rates between the awake and sleep conditions are causal for synaptic changes, but our previous work indicates that pairing synaptic inputs with ON‐states would weaken these inputs more strongly than pairing them with OFF‐states or waking activity (Bartram et al., 2017). To test this hypothesis, here we used an experimental paradigm modified from Vyazovskiy et al. (2008) to investigate whether pairing of electrical stimulation with ON and OFF periods during spontaneous NREM sleep leads to plastic changes in the motor cortex. Our preliminary data suggest that in the LFP, ON pairings significantly reduced the evoked responses compared with other conditions except OFF pairings. However, OFF pairings did not significantly reduce the peak amplitude. Hence, this dataset does not provide strong evidence that OFF pairings reduced the LFP response amplitude whereas ON pairings did so. The lack of significant difference between ON and OFF pairings could have to do with the incomplete separation between ON‐ and OFF‐states by the algorithm. Wake pairings were only significantly different compared with ON pairings, but showed a clear trend to increase the evoked response compared with other conditions. Indeed, the time of peak was significantly delayed by wake pairings, which could support the notion that wake pairing has a significant effect of its own. In the MUA, ON‐state pairings and wake pairings seemed to increase baseline firing rates. After correcting for changes in baseline firing, there was a significant effect of condition on the change in the mean number of evoked spikes. However, there was no evidence that ON‐state pairings led to a weakening. Overall, plasticity in the MUA appeared to be very subtle and did not show any conclusive directionality. We surmise that due to the well‐known variability between individual neurons (including the possibility that excitatory and inhibitory synapses are modulated in distinct ways; Bridi et al., 2019), the required sample size to observe an effect would have to be much larger in the MUA than in the LFP, and would possibly require single‐unit resolution.

Although our stimulation paradigm elicited only minor changes on sleep and SWA, future studies should consider the possibility of biologically significant effects of closed‐loop stimulation on other sleep characteristics, beyond merely sleep oscillations, as well as establish if there are any possible long‐term effects. If and how state‐specific stimulation modulates sleep is of importance because it could have an arousing (Segundo et al., 1955) or sleep‐promoting (Akert et al., 1952) effect, or alter sleep intensity (Landsness et al., 2011). To assess this, power spectral density was assessed in all 4‐s epochs that did not include a stimulation. This analysis revealed that OFF‐state pairings significantly decreased SWA activity compared with mock and ON‐state pairings, in a manner not linearly dependent on time in both the LFP and EEG. Furthermore, frequencies above approximately 11 Hz had increased power in the OFF‐ compared with ON‐state pairings. This strongly indicates a direct effect of OFF‐state pairings on sleep oscillations. The shift from lower to higher frequencies seems more consistent with arousal than with local changes in SWA. Our findings do not fully agree with previous studies using closed‐loop stimulation. For example, Ngo et al. (2013) calculated spectra across all 4‐s epochs during the pairing period, and found an increase in EEG SWA when auditory stimulation was targeted to the UP state and a decrease when the DOWN state was targeted. However, when epochs including stimulation were excluded from the latter analysis, the effect was no longer evident.

In summary, our study provides important new data demonstrating feasibility of in vivo targeting of neuronal OFF and ON periods in mice – the network counterparts of EEG or LFP slow waves. This method does not only represent a proof‐of‐concept that will inform translational studies, but it also establishes a new model for investigating the functional role of the slow oscillation in offline sensory processing and synaptic plasticity.

## AUTHOR CONTRIBUTIONS

EOM, MCK and VVV designed the study. MCK, LBK, CBD and MCCG conducted the experiments. MCK analysed the data. LBK and MCK performed histology. MCK and VVV wrote the manuscript with input from all authors.

## Data Availability

The data that support the findings of this study are available on request from the corresponding author.

## References

[jsr13603-bib-0001] Achermann, P. , Dijk, D. J. , Brunner, D. P. , & Borbely, A. A. (1993). A model of human sleep homeostasis based on EEG slow‐wave activity: Quantitative comparison of data and simulations. Brain Research Bulletin, 31(1–2), 97–113. 8453498 10.1016/0361-9230(93)90016-5

[jsr13603-bib-0002] Akert, K. , Koella, W. P. , & Hess, R., Jr. (1952). Sleep produced by electrical stimulation of the thalamus. The American Journal of Physiology, 168(1), 260–267. 10.1152/ajplegacy.1951.168.1.260 14903139

[jsr13603-bib-0003] Andrillon, T. , Burns, A. , Mackay, T. , Windt, J. , & Tsuchiya, N. (2021). Predicting lapses of attention with sleep‐like slow waves. Nature Communications, 12(1), 3657. 10.1038/s41467-021-23890-7 PMC8241869 34188023

[jsr13603-bib-0004] Bartram, J. , Kahn, M. C. , Tuohy, S. , Paulsen, O. , Wilson, T. , & Mann, E. O. (2017). Cortical up states induce the selective weakening of subthreshold synaptic inputs. Nature Communications, 8(1), 665. 10.1038/s41467-017-00748-5 PMC5610171 28939859

[jsr13603-bib-0005] Bellesi, M. , Riedner, B. A. , Garcia‐Molina, G. N. , Cirelli, C. , & Tononi, G. (2014). Enhancement of sleep slow waves: Underlying mechanisms and practical consequences. Frontiers in Systems Neuroscience, 8, 208. 10.3389/fnsys.2014.00208 25389394 PMC4211398

[jsr13603-bib-0006] Beltramo, R. , D'Urso, G. , Dal Maschio, M. , Farisello, P. , Bovetti, S. , Clovis, Y. , Lassi, G., Tucci, V., De Pietri Tonelli, D., & Fellin, T. (2013). Layer‐specific excitatory circuits differentially control recurrent network dynamics in the neocortex. Nature Neuroscience, 16, 227–234. 10.1038/nn.3306 23313909

[jsr13603-bib-0007] Bernardi, G. , Betta, M. , Ricciardi, E. , Pietrini, P. , Tononi, G. , & Siclari, F. (2019). Regional Delta waves in human rapid eye movement sleep. The Journal of Neuroscience, 39(14), 2686–2697. 10.1523/JNEUROSCI.2298-18.2019 30737310 PMC6445986

[jsr13603-bib-0008] Besedovsky, L. , Ngo, H. V. , Dimitrov, S. , Gassenmaier, C. , Lehmann, R. , & Born, J. (2017). Auditory closed‐loop stimulation of EEG slow oscillations strengthens sleep and signs of its immune‐supportive function. Nature Communications, 8(1), 1984. 10.1038/s41467-017-02170-3 PMC5719447 29215045

[jsr13603-bib-0009] Borbély, A. A. (1982). A two process model of sleep regulation. Human Neurobiology, 1(3), 195–204. 7185792

[jsr13603-bib-0010] Borbely, A. A. , Tobler, I. , & Hanagasioglu, M. (1984). Effect of sleep deprivation on sleep and EEG power spectra in the rat. Behavioural Brain Research, 14(3), 171–182. 10.1016/0166-4328(84)90186-4 6525241

[jsr13603-bib-0011] Bridi, M. C. D. , Zong, F. J. , Min, X. , Luo, N. , Tran, T. , Qiu, J. , Severin, D., Zhang, X. T., Wang, G., Zhu, Z. J., He, K. W., & Kirkwood, A. (2019). Daily oscillation of the excitation‐inhibition balance in visual cortical circuits. Neuron, 105, 621–629.e4. 10.1016/j.neuron.2019.11.011 31831331 PMC9520672

[jsr13603-bib-0012] Bruning, F. , Noya, S. B. , Bange, T. , Koutsouli, S. , Rudolph, J. D. , Tyagarajan, S. K. , Cox, J., Mann, M., Brown, S. A., & Robles, M. S. (2019). Sleep‐wake cycles drive daily dynamics of synaptic phosphorylation. Science, 366(6462), eaav3617. 10.1126/science.aav3617 31601740

[jsr13603-bib-0013] Bukhtiyarova, O. , Soltani, S. , Chauvette, S. , & Timofeev, I. (2019). Slow wave detection in sleeping mice: Comparison of traditional and machine learning methods. Journal of Neuroscience Methods, 316, 35–45. 10.1016/j.jneumeth.2018.08.016 30125590

[jsr13603-bib-0014] Chaure, F. J. , Rey, H. G. , & Quian Quiroga, R. (2018). A novel and fully automatic spike‐sorting implementation with variable number of features. Journal of Neurophysiology, 120(4), 1859–1871. 10.1152/jn.00339.2018 29995603 PMC6230803

[jsr13603-bib-0015] Chauvette, S. , Seigneur, J. , & Timofeev, I. (2012). Sleep oscillations in the thalamocortical system induce long‐term neuronal plasticity. Neuron, 75(6), 1105–1113. 10.1016/j.neuron.2012.08.034 22998877 PMC3458311

[jsr13603-bib-0016] Choi, J. , Kwon, M. , & Jun, S. C. (2020). A systematic review of closed‐loop feedback techniques in sleep studies‐related issues and future directions. Sensors (Basel), 20(10), 2770. 10.3390/s20102770 PMC7285770 32414060

[jsr13603-bib-0017] Chung, J. E. , Magland, J. F. , Barnett, A. H. , Tolosa, V. M. , Tooker, A. C. , Lee, K. Y. , Shah, K. G., Felix, S. H., Frank, L. M., & Greengard, L. F. (2017). A fully automated approach to spike sorting. Neuron, 95(6), 1381–1394 e1386. 10.1016/j.neuron.2017.08.030 28910621 PMC5743236

[jsr13603-bib-0018] Cirelli, C. (2017). Sleep, synaptic homeostasis and neuronal firing rates. Current Opinion in Neurobiology, 44, 72–79. 10.1016/j.conb.2017.03.016 28399462 PMC5605801

[jsr13603-bib-0019] de Vivo, L. , Bellesi, M. , Marshall, W. , Bushong, E. A. , Ellisman, M. H. , Tononi, G. , & Cirelli, C. (2017). Ultrastructural evidence for synaptic scaling across the wake/sleep cycle. Science, 355(6324), 507–510. 10.1126/science.aah5982 28154076 PMC5313037

[jsr13603-bib-0020] Diering, G. H. S. N. R. , Roth, R. H. , Worley, P. F. , Pandey, A. , & Huganir, R. L. (2017). Homer1a drives homeostatic scaling‐down of excitatory synapses during sleep. Science, 355(6324), 511–515. 28154077 10.1126/science.aai8355 PMC5382711

[jsr13603-bib-0021] Fattinger, S. , Heinzle, B. B. , Ramantani, G. , Abela, L. , Schmitt, B. , & Huber, R. (2019). Closed‐loop acoustic stimulation during sleep in children with epilepsy: A hypothesis‐driven novel approach to interact with spike‐wave activity and pilot data assessing feasibility. Frontiers in Human Neuroscience, 13, 166. 10.3389/fnhum.2019.00166 31164813 PMC6536690

[jsr13603-bib-0022] Fisher, S. P. , Cui, N. , McKillop, L. E. , Gemignani, J. , Bannerman, D. M. , Oliver, P. L. , & Vyazovskiy, V. V. (2016). Stereotypic wheel running decreases cortical activity in mice. Nature Communications, 7, 13138. 10.1038/ncomms13138 PMC5071642 27748455

[jsr13603-bib-0023] Frank, M. G. , & Heller, H. C. (2019). The function(s) of sleep. Handbook of Experimental Pharmacology, 253, 3–34. 10.1007/164_2018_140 31004225

[jsr13603-bib-0024] Frase, L. , Selhausen, P. , Krone, L. , Tsodor, S. , Jahn, F. , Feige, B. , Maier, J. G., Mainberger, F., Piosczyk, H., Kuhn, M., Kloppel, S., Sterr, A., Baglioni, C., Spiegelhalder, K., Riemann, D., Nitsche, M. A., & Nissen, C. (2019). Differential effects of bifrontal tDCS on arousal and sleep duration in insomnia patients and healthy controls. Brain Stimulation, 12(3), 674–683. 10.1016/j.brs.2019.01.001 30639236

[jsr13603-bib-0025] Funk, C. M. , Honjoh, S. , Rodriguez, A. V. , Cirelli, C. , & Tononi, G. (2016). Local slow waves in superficial layers of primary cortical areas during REM sleep. Current Biology, 26(3), 396–403. 26804554 10.1016/j.cub.2015.11.062 PMC4747819

[jsr13603-bib-0026] Geiser, T. , Hertenstein, E. , Feher, K. , Maier, J. G. , Schneider, C. L. , Zust, M. A. , … Nissen, C. (2020). Targeting arousal and sleep through noninvasive brain stimulation to improve mental health. Neuropsychobiology, 79(4–5), 284–292. 10.1159/000507372 32408296

[jsr13603-bib-0027] Gonzalez‐Rueda, A. , Pedrosa, V. , Feord, R. C. , Clopath, C. , & Paulsen, O. (2018). Activity‐dependent downscaling of subthreshold synaptic inputs during slow‐wave‐sleep‐like activity in Vivo. Neuron, 97(6), 1244–1252 e1245. 10.1016/j.neuron.2018.01.047 29503184 PMC5873548

[jsr13603-bib-0069] Haider, B., Duque, A., Hasenstaub, A. R., Yu Y., & McCormick, D. A. (2007). Enhancement of visual responsiveness by spontaneous local networkactivity in vivo. Journal of Neurophysiology, 97(6), 4186–4202. 10.1152/jn.01114.2006 17409168

[jsr13603-bib-0028] Harrison, X. A. , Donaldson, L. , Correa‐Cano, M. E. , Evans, J. , Fisher, D. N. , Goodwin, C. E. D. , Robinson, B. S., Hodgson, D. J., & Inger, R. (2018). A brief introduction to mixed effects modelling and multi‐model inference in ecology. PeerJ, 6, e4794. 10.7717/peerj.4794 29844961 PMC5970551

[jsr13603-bib-0029] Hengen, K. B. , Torrado Pacheco, A. , McGregor, J. N. , Van Hooser, S. D. , & Turrigiano, G. G. (2016). Neuronal firing rate homeostasis is inhibited by sleep and promoted by wake. Cell, 165(1), 180–191. 10.1016/j.cell.2016.01.046 26997481 PMC4809041

[jsr13603-bib-0030] Huber, R. , Deboer, T. , & Tobler, I. (2000). Effects of sleep deprivation on sleep and sleep EEG in three mouse strains: Empirical data and simulations. Brain Research, 857(1–2), 8–19. 10700548 10.1016/s0006-8993(99)02248-9

[jsr13603-bib-0031] Krone, L. B. , Yamagata, T. , Blanco‐Duque, C. , Guillaumin, M. C. C. , Kahn, M. C. , van der Vinne, V. , McKillop, L. E., Tam, S. K. E., Peirson, S. N., Akerman, C. J., Hoerder‐Suabedissen, A., Molnar, Z., Vyazovskiy, V. V. (2021). A role for the cortex in sleep‐wake regulation. Nature Neuroscience, 24, 1210–1215. 10.1038/s41593-021-00894-6 34341585 PMC7612118

[jsr13603-bib-0032] Krueger, J. M. , Frank, M. G. , Wisor, J. P. , & Roy, S. (2016). Sleep function: Toward elucidating an enigma. Sleep Medicine Reviews, 28, 42–50. 10.1016/j.smrv.2015.08.005 PMC4769986 26447948

[jsr13603-bib-0033] Krueger, J. M. , Rector, D. M. , Roy, S. , Van Dongen, H. P. , Belenky, G. , & Panksepp, J. (2008). Sleep as a fundamental property of neuronal assemblies. Nature Reviews. Neuroscience, 9(12), 910–919. 10.1038/nrn2521 18985047 PMC2586424

[jsr13603-bib-0034] Krugliakova, E. , Skorucak, J. , Sousouri, G. , Leach, S. , Snipes, S. , Ferster, M. L. , Da Poian, G., Karlen, W., & Huber, R. (2022). Boosting recovery during sleep by means of auditory stimulation. Frontiers in Neuroscience, 16. 10.3389/fnins.2022.755958 PMC8847378 35185455

[jsr13603-bib-0035] Landsness, E. C. , Goldstein, M. R. , Peterson, M. J. , Tononi, G. , & Benca, R. M. (2011). Antidepressant effects of selective slow wave sleep deprivation in major depression: A high‐density EEG investigation. Journal of Psychiatric Research, 45(8), 1019–1026. 10.1016/j.jpsychires.2011.02.003 21397252 PMC3119746

[jsr13603-bib-0036] Malkani, R. G. , & Zee, P. C. (2020). Brain stimulation for improving sleep and memory. Sleep Medicine Clinics, 15(1), 101–115. 10.1016/j.jsmc.2019.11.002 32005347

[jsr13603-bib-0037] Marshall, L. , Helgadottir, H. , Molle, M. , & Born, J. (2006). Boosting slow oscillations during sleep potentiates memory. Nature, 444(7119), 610–613. 10.1038/nature05278 17086200

[jsr13603-bib-0038] Massimini, M. , Ferrarelli, F. , Huber, R. , Esser, S. K. , Singh, H. , & Tononi, G. (2005). Breakdown of cortical effective connectivity during sleep. Science, 309(5744), 2228–2232. 16195466 10.1126/science.1117256

[jsr13603-bib-0039] Massimini, M. , Huber, R. , Ferrarelli, F. , Hill, S. , & Tononi, G. (2004). The sleep slow oscillation as a traveling wave. The Journal of Neuroscience, 24(31), 6862–6870. 15295020 10.1523/JNEUROSCI.1318-04.2004 PMC6729597

[jsr13603-bib-0040] McKillop, L. E. , Fisher, S. P. , Cui, N. , Peirson, S. N. , Foster, R. G. , Wafford, K. A. , & Vyazovskiy, V. V. (2018). Effects of aging on cortical neural dynamics and local sleep homeostasis in mice. The Journal of Neuroscience, 38(16), 3911–3928. 10.1523/JNEUROSCI.2513-17.2018 29581380 PMC5907054

[jsr13603-bib-0071] Mitra, P., & Bokil, H. (2008). Observed brain dynamics. Oxford University Press. p. 381.

[jsr13603-bib-0041] Moreira, C. G. , Baumann, C. R. , Scandella, M. , Nemirovsky, S. I. , Leach, S. , Huber, R. , & Noain, D. (2021). Closed‐loop auditory stimulation method to modulate sleep slow waves and motor learning performance in rats. eLife, 10, e68043. 10.7554/eLife.68043 PMC8530509 34612204

[jsr13603-bib-0042] Murphy, M. , Riedner, B. A. , Huber, R. , Massimini, M. , Ferrarelli, F. , & Tononi, G. (2009). Source modeling sleep slow waves. Proceedings of the National Academy of Sciences of the United States of America, 106(5), 1608–1613. 10.1073/pnas.0807933106 19164756 PMC2635823

[jsr13603-bib-0043] Ngo, H. V. , Martinetz, T. , Born, J. , & Molle, M. (2013). Auditory closed‐loop stimulation of the sleep slow oscillation enhances memory. Neuron, 78, 545–553. 10.1016/j.neuron.2013.03.006 23583623

[jsr13603-bib-0044] Nir, Y. , Andrillon, T. , Marmelshtein, A. , Suthana, N. , Cirelli, C. , Tononi, G. , & Fried, I. (2017). Selective neuronal lapses precede human cognitive lapses following sleep deprivation. Nature Medicine, 23(12), 1474–1480. 10.1038/nm.4433 PMC5720899 29106402

[jsr13603-bib-0045] Nir, Y. , Staba, R. J. , Andrillon, T. , Vyazovskiy, V. V. , Cirelli, C. , Fried, I. , & Tononi, G. (2011). Regional slow waves and spindles in human sleep. Neuron, 70(1), 153–169. 10.1016/j.neuron.2011.02.043 21482364 PMC3108825

[jsr13603-bib-0046] Nir, Y. , Vyazovskiy, V. V. , Cirelli, C. , Banks, M. I. , & Tononi, G. (2015). Auditory responses and stimulus‐specific adaptation in rat auditory cortex are preserved across NREM and REM sleep. Cerebral Cortex, 25(5), 1362–1378. 10.1093/cercor/bht328 24323498 PMC4415088

[jsr13603-bib-0047] Noya, S. B. , Colameo, D. , Bruning, F. , Spinnler, A. , Mircsof, D. , Opitz, L. , Mann, M., Tyagarajan, S. K., Robles, M. S., Brown, S. A. (2019). The forebrain synaptic transcriptome is organized by clocks but its proteome is driven by sleep. Science, 366(6462), eaav2642. 10.1126/science.aav2642 31601739

[jsr13603-bib-0048] Paxinos, G. , & Franklin, K. B. J. (2001). The mouse brain in stereotaxic coordinates (2nd ed.). Academic Press.

[jsr13603-bib-0049] Petreanu, L. , Huber, D. , Sobczyk, A. , & Svoboda, K. (2007). Channelrhodopsin‐2‐assisted circuit mapping of long‐range callosal projections. Nature Neuroscience, 10(5), 663–668. 10.1038/nn1891 17435752

[jsr13603-bib-0070] Reig, R., Zerlaut, Y., Vergara, R., Destexhe, A., & Sanchez‐Vives, M. V. (2015) Gain modulation of synaptic inputs by network state in auditorycortex in vivo. Journal of Neurophysiology, 35(6), 2689–2702. 10.1523/JNEUROSCI.2004-14.2015 PMC6605611 25673859

[jsr13603-bib-0050] Riedner, B. A. , Hulse, B. K. , Murphy, M. J. , Ferrarelli, F. , & Tononi, G. (2011). Temporal dynamics of cortical sources underlying spontaneous and peripherally evoked slow waves. Progress in Brain Research, 193, 201–218. 10.1016/B978-0-444-53839-0.00013-2 21854964 PMC3160723

[jsr13603-bib-0051] Sanchez‐Vives, M. V. , & McCormick, D. A. (2000). Cellular and network mechanisms of rhythmic recurrent activity in neocortex. Nature Neuroscience, 3(10), 1027–1034. 11017176 10.1038/79848

[jsr13603-bib-0052] Santostasi, G. , Malkani, R. , Riedner, B. , Bellesi, M. , Tononi, G. , Paller, K. A. , & Zee, P. C. (2016). Phase‐locked loop for precisely timed acoustic stimulation during sleep. Journal of Neuroscience Methods, 259, 101–114. 10.1016/j.jneumeth.2015.11.007 26617321 PMC5169172

[jsr13603-bib-0053] Schneider, J. , Lewis, P. A. , Koester, D. , Born, J. , & Ngo, H. V. (2020). Susceptibility to auditory closed‐loop stimulation of sleep slow oscillations changes with age. Sleep, 43(12), zsaa111. 10.1093/sleep/zsaa111 PMC7734479 32562487

[jsr13603-bib-0054] Segundo, J. P. , Naquet, R. , & Buser, P. (1955). Effects of cortical stimulation on electro‐cortical activity in monkeys. Journal of Neurophysiology, 18(3), 236–245. 10.1152/jn.1955.18.3.236 14368335

[jsr13603-bib-0055] Seibt, J. , & Frank, M. G. (2019). Primed to sleep: The dynamics of synaptic plasticity across brain states. Frontiers in Systems Neuroscience, 13, 2. 10.3389/fnsys.2019.00002 30774586 PMC6367653

[jsr13603-bib-0056] Siclari, F. , & Tononi, G. (2017). Local aspects of sleep and wakefulness. Current Opinion in Neurobiology, 44, 222–227. 28575720 10.1016/j.conb.2017.05.008 PMC6445546

[jsr13603-bib-0057] Skoglund, T. S. , Pascher, R. , & Berthold, C. H. (1997). The existence of a layer IV in the rat motor cortex. Cerebral Cortex, 7(2), 178–180. 10.1093/cercor/7.2.178 9087825

[jsr13603-bib-0058] Thomas, C. W. , Guillaumin, M. C. , McKillop, L. E. , Achermann, P. , & Vyazovskiy, V. V. (2020). Global sleep homeostasis reflects temporally and spatially integrated local cortical neuronal activity. eLife, 9, e54148. 10.7554/eLife.54148 PMC7332296 32614324

[jsr13603-bib-0059] Timofeev, I. (2013). Local origin of slow EEG waves during sleep. Zhurnal Vyssheĭ Nervnoĭ Deiatelnosti Imeni I P Pavlova, 63(1), 105–112. 23697226 10.7868/s0044467713010139

[jsr13603-bib-0060] Vyazovskiy, V. V. , Cirelli, C. , Pfister‐Genskow, M. , Faraguna, U. , & Tononi, G. (2008). Molecular and electrophysiological evidence for net synaptic potentiation in wake and depression in sleep. Nature Neuroscience, 11(2), 200–208. 10.1038/nn2035 18204445

[jsr13603-bib-0061] Vyazovskiy, V. V. , Cui, N. , Rodriguez, A. V. , Funk, C. , Cirelli, C. , & Tononi, G. (2014). The dynamics of cortical neuronal activity in the first minutes after spontaneous awakening in rats and mice. Sleep, 37(8), 1337–1347. 10.5665/sleep.3926 25083014 PMC4096203

[jsr13603-bib-0062] Vyazovskiy, V. V. , Faraguna, U. , Cirelli, C. , & Tononi, G. (2009). Triggering slow waves during NREM sleep in the rat by intracortical electrical stimulation: Effects of sleep/wake history and background activity. Journal of Neurophysiology, 101(4), 1921–1931. 10.1152/jn.91157.2008 19164101 PMC2695630

[jsr13603-bib-0063] Vyazovskiy, V. V. , & Harris, K. D. (2013). Sleep and the single neuron: The role of global slow oscillations in individual cell rest. Nature Reviews. Neuroscience, 14(6), 443–451. 10.1038/nrn3494 23635871 PMC3972489

[jsr13603-bib-0064] Vyazovskiy, V. V. , Olcese, U. , Cirelli, C. , & Tononi, G. (2013). Prolonged wakefulness alters neuronal responsiveness to local electrical stimulation of the neocortex in awake rats. Journal of Sleep Research, 22(3), 239–250. 10.1111/jsr.12009 23607417 PMC3723708

[jsr13603-bib-0065] Vyazovskiy, V. V. , Olcese, U. , Hanlon, E. C. , Nir, Y. , Cirelli, C. , & Tononi, G. (2011). Local sleep in awake rats. Nature, 472(7344), 443–447. 10.1038/nature10009 21525926 PMC3085007

[jsr13603-bib-0066] Vyazovskiy, V. V. , Olcese, U. , Lazimy, Y. M. , Faraguna, U. , Esser, S. K. , Williams, J. C. , Cirelli, C., & Tononi, G. (2009). Cortical firing and sleep homeostasis. Neuron, 63(6), 865–878. 10.1016/j.neuron.2009.08.024 19778514 PMC2819325

[jsr13603-bib-0067] Watson, B. O. , Levenstein, D. , Greene, J. P. , Gelinas, J. N. , & Buzsaki, G. (2016). Network homeostasis and state dynamics of neocortical sleep. Neuron, 90(4), 839–852. 10.1016/j.neuron.2016.03.036 27133462 PMC4873379

[jsr13603-bib-0068] Yamawaki, N. , Borges, K. , Suter, B. A. , Harris, K. D. , & Shepherd, G. M. (2014). A genuine layer 4 in motor cortex with prototypical synaptic circuit connectivity. eLife, 3, e05422. 10.7554/eLife.05422 25525751 PMC4290446

